# Long-term HbA1c variability and macro-/micro-vascular complications in type 2 diabetes mellitus: a meta-analysis update

**DOI:** 10.1007/s00592-023-02037-8

**Published:** 2023-01-30

**Authors:** Giovanni Sartore, Eugenio Ragazzi, Rosaria Caprino, Annunziata Lapolla

**Affiliations:** 1 Department of Medicine – DIMED, University of Padua, Padua, Italy; 2 Department of Pharmaceutical and Pharmacological Sciences – DSF, University of Padua, Padua, Italy

**Keywords:** HbA1c variability, Type 2 diabetes mellitus, Macro-vascular and micro-vascular complications, Meta-analysis

## Abstract

**Aims:**

The aim of the present study was to evaluate, by means of a meta-analysis approach, whether new available data, appeared on qualified literature, can support the effectiveness of an association of HbA1c variability with the risk of macro- and/or micro-vascular complications in type 2 diabetes mellitus (T2DM).

**Methods:**

The meta-analysis was conducted according to PRISMA Statement guidelines and considered published studies on T2DM, presenting HbA1c variability as standard deviation (SD) or its derived coefficient of variation (CV). Literature search was performed on PubMed in the time range 2015–July 2022, with no restrictions of language.

**Results:**

Twenty-three selected studies fulfilled the aims of the present investigation. Overall, the analysis of the risk as hazard ratios (HR) indicated a significant association between the HbA1c variability, expressed either as SD or CV, and the complications, except for neuropathy. Macro-vascular complications were all significantly associated with HbA1c variability, with HR 1.40 (95%CI 1.31–1.50, *p* < 0.0001) for stroke, 1.30 (95%CI 1.25–1.36, *p* < 0.0001) for transient ischaemic attack/coronary heart disease/myocardial infarction, and 1.32 (95%CI 1.13–1.56, *p* = 0.0007) for peripheral arterial disease. Micro-vascular complications yielded HR 1.29 (95%CI 1.22–1.36, *p* < 0.0001) for nephropathy, 1.03 (95%CI 0.99–1.08, *p* = 0.14) for neuropathy, and 1.15 (95%CI 1.08–1.24, *p* < 0.0001) for retinopathy. For all-cause mortality, HR was 1.33 (95%CI 1.27–1.39, *p* < 0.0001), and for cardiovascular mortality 1.25 (95%CI 1.17–1.34, *p* < 0.0001).

**Conclusions:**

Our meta-analysis on HbA1c variability performed on the most recent published data since 2015 indicates positive association between HbA1c variability and macro-/micro-vascular complications, as well as mortality events, in T2DM, suggesting that this long-term glycaemic parameter merits further attention as a predictive, independent risk factor for T2DM population.

**Supplementary Information:**

The online version contains supplementary material available at 10.1007/s00592-023-02037-8.

## Introduction

Glycated haemoglobin (HbA1c) is a biomarker with central role in the diagnosis and follow-up of patients with diabetes mellitus, although not a perfect one [1]. Since first discovery of HbA1c in the late 1960s, its use as marker of glycaemic control has gradually increased over the course of the last four decades [2]. American Diabetes Association (ADA) recommends HbA1c determination in patients with diabetes mellitus on therapy in order to monitor the glycometabolic status in the medium–long term and thus reduce the risk of vascular complications [3]. Previous large-scale clinical trials, such as the UKPDS (United Kingdom Prospective Diabetes Study) and ADVANCE (Action in Diabetes and Vascular Disease: Preterax and Diamicron-MR controlled evaluation), have reported a significant reduction in mortality and cardiovascular complications by tighter glycaemic control, excluded patients with major comorbidities [4–6]. Studies showed that even slight elevations of HbA1c concentration in blood correlated with an increased cardiovascular risk [7, 8]. However, there is evidence for increased mortality risk for patients in both extremes of HbA1c, so that the 2008 Action to Control Cardiovascular Risk in Diabetes (ACCORD) trial was ended prematurely due to significantly higher mortality reported in the intensive glycaemic control group [9].

Research has been exploring other parameters that can offer more accurate and individualized disease monitoring. Glycaemic variability can be measured over the short term with continue glucose monitoring (CGM) of interstitial glucose levels both within-day and between-day, and it can also be assessed over the long term (months to years) by the oscillation of fasting plasma glucose or HbA1c levels. Long-term glycaemic variability is most commonly assessed by HbA1c variability [10], which can be used as a predictor for complications and mortality, inasmuch increased HbA1c variability has been associated with diabetic complications in various organ systems, in addition to all-cause and cardiovascular mortality [10–12]. However, the clinical association between long-term glycaemic variability and diabetes complications is difficult to establish because of heterogeneity among studies, including their design and the different metrics used to assess the glycaemic variability.

Traditional measures of HbA1c variability can include the standard deviation (SD) and its derived coefficient of variation (CV), the latter calculated as SD divided by the mean [13]. However, neither SD nor CV of HbA1c can be easily interpreted in clinical practice, and moreover, they only reflect the dispersion of the measurements around a single value (the mean) not considering the order of the measurements obtained [13]. Bonke et al. [14] argued that SD has two main problems. First, the length of time between measurements is ignored, leading to potentially misleading conclusions when HbA1c measurements are widely spaced. Second, with only a small number of measurements per patient, the validity and interpretation of the standard deviation, even with correction, are limited.

The systematic review and meta-analysis provided by Gorst et al. [15] have suggested that HbA1c variability is positively associated with the risk of micro- and macro-vascular complications in type 1 diabetes mellitus (T1DM) and type 2 diabetes mellitus (T2DM) patients, independently of the HbA1c level. The study was based on the analysis of 20 published reports in a ten-year time interval between 2004 and 2014. Authors, however, highlighted that most studies were retrospective, therefore lacking adjustment for confounders, and inconsistency was found in the definition of HbA1c variability. They suggested that further studies on the relationship between HbA1c variability and diabetes complications are needed to confirm the relevance of this measure as a risk prediction for diabetes-related negative outcomes.

The aim of the present study was to evaluate, by means of a meta-analysis approach, whether new available data, appeared on qualified literature since 2015, can support the effectiveness of an association of HbA1c variability with the risk of macro- and/or micro-vascular complications in T2DM.

## Methods

The meta-analysis was conducted according to PRISMA Statement guidelines [16], following the suggested checklist of items.

### Parameters of interest for HbA1c variability

As anticipated in Introduction, traditional measures of HbA1c variability include the standard deviation (SD) and the derived coefficient of variation (CV), calculated as SD divided by the mean [13]. Since the number of HbA1c measurements can influence SD value (e.g. fewer measurements making the SD greater), several studies calculate an SD value adjusted for the number of HbA1c measurements, defined according to the formula: adjusted HbA1c SD = SD/√[n/(n − 1)] [17]. Other methods suggested to calculate variation independent of the mean (VIM), average real variability (ARV), or average successive variability (ASV), which is the average absolute difference between successive values. VIM was defined as the SD divided by the mean to the power x and multiplied by the population mean to the power x, with x derived from curve fitting and ARV as the average of the absolute differences between consecutive HbA1c measurements [18]. Recently, Forbes et al. [11] developed a new scale, namely the HbA1c variability score (HVS), indicating how frequently HbA1c rises or decreases by > 0.5% (5.5 mmol/mol), which is in line with the SD and CV of HbA1c but clinically more translatable. Bonke et al. [14] defined HbA1c variability using the difference between successive measurements.

The present meta-analysis considered published studies presenting SD and CV, since these parameters are the most commonly available indices for HbA1c variability.

### Data sources and searches

Literature search was performed on PubMed in the time range 2015–July 2022, with no restrictions of language, using as search terms the followings:

(HbA1c variability OR glycosylated haemoglobin variation OR HbA1c CV OR HbA1c SD OR HbA1c coefficient of variation OR HbA1c standard variation) AND (type 2 diabetes mellitus AND (microvascular complications OR macrovascular complications)).

All resulting articles were reviewed by two reviewers (R.C. and G.S.).

### Selection of studies

Eligibility criteria were selected according to the PICOS framework [19, 20]:

- P: Population: patients (age > 18y) with diagnosis of T2DM;
- I: Investigated condition: measurement of HbA1c variability, assessed by the SD or CV;
- C: Comparison condition: logistic or Cox regression analysis for outcome risk prediction;
- O: Outcome: risk of adverse macro-/micro-vascular complications;
- S: Study type: any kinds of clinical trials (randomized controlled trial, cohort study, etc.).

All the articles that fulfilled the requirements were considered, without restriction on ages of participants (as long as they were adults). We excluded reviews, editorials, and case reports. Full articles on potentially relevant studies were downloaded and reviewed for inclusion.

The main adverse outcomes of interest were all-cause mortality, cardiovascular mortality, and both diabetes macro-complications (stroke; TIA: transient ischaemic attack; CHD: coronary heart disease; MI: myocardial infarction; PAD: peripheral arterial disease) and micro-complications (nephropathy, neuropathy, retinopathy). When an article presented more estimates of the HbA1c variability, based on different models, all the proposed data were considered.

### Data analysis

Meta-analysis was performed using review manager (RevMan) [computer program] version 5.4.1 (The Cochrane Collaboration, 2020). Analysis was stratified according to the presence of data regarding HbA1c variability in terms of coefficient of variation (CV) or as standard deviation (SD) and using hazard ratios (HR) or odds ratios (OR) data, as made available by considered studies. Subgroup analyses and overall values were presented. Separate analyses of HR and OR were performed, due to the different nature of the risk parameter meaning. Analysis was performed using the random-effects method [21, 22]. Results are graphically presented as forest plots, according to inverse-variance approach. Data were entered into RevMan as natural logarithm of the risk parameter with its standard error (as natural logarithm). Conversion of confidence intervals (CI) to standard error was obtained with the formula (ln upper CI−ln lower CI)/(2 × 1.96).

The measure of the extent of variation (heterogeneity) among the effects observed in different studies was quantified by Tau^2^. Heterogeneity was also evaluated by using *I*^*2*^ statistics based on *χ*^2^ test [23] considering the following suggested levels of heterogeneity: 0–40% might not be important; 30–60% may represent moderate heterogeneity; 50–90% may represent substantial heterogeneity; 75–100% may represent considerable heterogeneity. Test for overall effect for each group and across all subgroups was executed based on *z*-distribution and significance results provided [24].

## Results

**Fig. 1:**
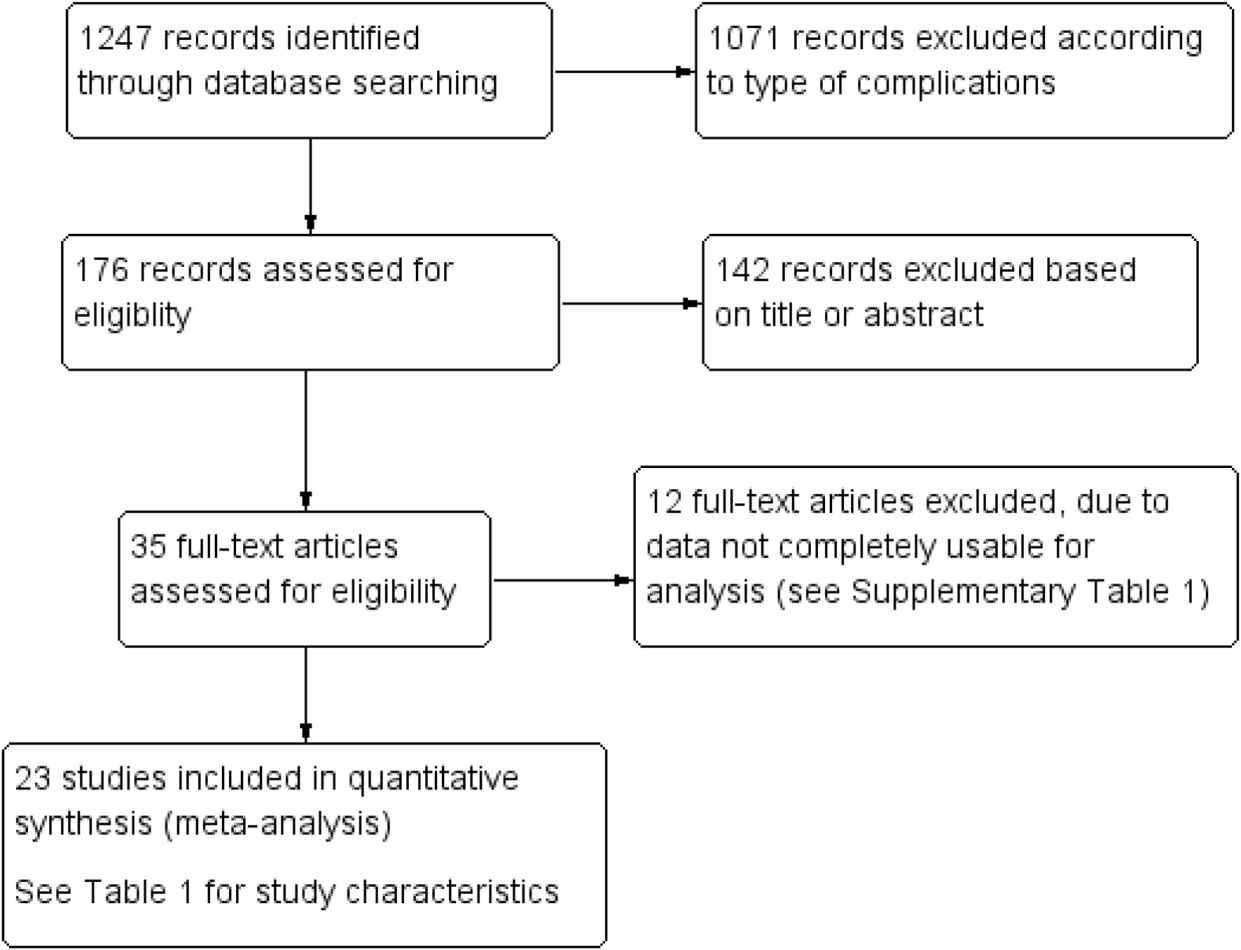
Flowchart of study selection

Figure 1 presents the flowchart of the details of study selection performed in this meta-analysis. Of the initial 1247 records identified on the topic HbA1c variability in T2DM, after filtering for complications, 176 records were selected, and 35 potentially relevant studies were identified. Of these, following full-text screening, 23 studies fulfilled the aims of the present investigation and were used for quantitative synthesis through meta-analysis. Table 1 illustrates the prominent characteristics of the studies considered in the meta-analysis (Table S1 reports the characteristics of the 12 excluded articles, which, although containing pertinent data, were not usable for the present analysis).

**Table 1:** Characteristics of the studies considered in the meta-analysis. Studies are presented in chronological order of publication

Study	Type of study; time interval considered; country	Sample size (n)	Age of patients (mean ± SD, or median & range)	Gender (Male %)	Inclusion criteria	Measure of HbA1c variability	Methodological notes	Evaluated complications
Takao et al., 2015 [25]	Retrospective study; Jan 1995–Dec 1996;	632	56 ± 9	82	Patients with T2DM who first visited the outpatient clinic	CV	HR from multivariate analysis using Cox proportional hazard models	Any CVD event
Prentice et al., 2016 [26]	Retrospective cohort study from veterans’ health administration; 2000–2009; USA	50 861	66 ± 9	98	Individuals with T2DM	SD; CV	HR from Cox proportional hazards models	All-cause mortality
Takenouchi et al., 2016 [27]	Prospective cohort study; 2004–2005; Japan	162	62 ± 10	55	Patients with T2DM and preserved kidney function	CV	HR from Cox proportional hazard regression models	Diabetic nephropathy, deterioration of chronic kidney disease
Wan et al., 2016 [28]	Population-based retrospective cohort study; Aug 2008–Dec 2009; Hong Kong	91,866	63 ± 11	44	Patients aged 18 years or above, clinically diagnosed with T2DM without prior history of CVD	SD; CV	HR from multivariable Cox proportional hazards regression	CVD and all-cause mortality
Dorajoo et al., 2017 [29]	Retrospective cohort study; 2010–2014; Singapore	716	56 ± 13	52	Patients with T2DM attending outpatient diabetes centre	CV	OR from multivariate logistic regression	Diabetic nephropathy
Foo et al., 2017 [30]	Retrospective case–control study; 2012–2013; South Singapore	172 + 172	61 + 11	75	Patients with T2DM who had undergone retinal screening at a public primary care clinic	SD	OR from sex-adjusted logistic regression analysis, 2 models	Diabetic retinopathy
Jun et al., 2017 [31]	Retrospective longitudinal study; Sept 2011–March 2017; South Korea	498	59 ± 10	60	Patients with T2DM without cardiovascular autonomic neuropathy (CAN)	SD; CV	OR from multivariate logistic regression analysis, 3 models	Cardiovascular autonomic neuropathy
Lee M.Y. et al., 2017 [32]	Longitudinal study on the Kaohsiung Medical University research database; 2009–2015; Taiwan	8259	62 ± 12	52	Patients with T2DM	SD	HR from Cox proportional hazard regression analysis, univariate and multivariate models	Multiple cardiovascular events
Takao et al., 2017 [33]	Retrospective observational cohort study; 1995–2012; Japan	832	55 ± 10	82	Patients with T2DM attending outpatient clinic	CV	HR from multivariate analyses using Cox proportional hazard models	Nephropathy and retinopathy
Cardoso et al., 2018 [34]	Prospective cohort study, nested within The Rio de Janeiro type 2 diabetes cohort study; Aug 2004–Dec 2008 re-evaluated annually until Dec 2016; Brazil	654	60 ± 10	38	Adult type T2DM individual up to 80 years old with either any micro-vascular (retinopathy, nephropathy or neuropathy) or macro-vascular (coronary, cerebrovascular or peripheral artery disease) complication	SD; CV	Cox survival analyses with different models for HR determination	Micro- and macro-vascular complications, and all-cause mortality
Su et al., 2018 [12]	Cross-sectional observational study; Feb 2011–Dec 2016; China	563	56 ± 10	53	Patients with T2DM	CV	Odds ratios with 5 models	Diabetic neuropathy
Zhou et al., 2018 [35]	Retrospective study based on veterans affairs diabetes trial (VADT); –; USA	1 791	60	97	Military veterans with suboptimal response to therapy for T2DM	CV	HR from Cox proportional hazard models	Cardiovascular disease (CVD)
Critchley et al., 2019 [36]	Retrospective matched cohort study based on English primary care database (Clinical Practice Research Datalink, CPRD); 2006–2009; UK	58 832	68 ± 11	55	Patients who were identified as having T2DM	CV	HR from Cox regression	All-cause mortality, cardiovascular (CVD) mortality
Ceriello et al., 2020 [37]	EMPA-REG OUTCOME; 2010–2013; Europe, North America, Asia	7034	63.1	72	Patients with T2DM, randomized to receive placebo or empagliflozin	SD; CV	HR from Cox proportional hazard models	Cardiovascular (CVD) mortality
Scott et al., 2020 [38]	Randomized placebo-controlled trial on fenofibrate (FIELD study); median 5y; 63 centres in Australia, New Zealand, and Finland	9795	50–75	63	People with T2DM randomized to 200 mg fenofibrate or placebo	SD; CV	Cox proportional hazards regression analyses; 3 models with adjustment for pre-specified variables	Micro- and macro-vascular complications and mortality
Sheng et al., 2020 [18]	Multicentre clinical study “Action to Control Cardiovascular Risk in Diabetes” (ACCORD) trial; 3 y; USA, Canada	9483	63 ± 7	62	Patients with T2DM enrolled in the Action to Control Cardiovascular Risk in Diabetes (ACCORD) trial	CV	Hazard ratio (HR) from Cox regression models	All-cause mortality
Wan et al., 2020 [39]	Prospective cohort study; Jan 2008–Dec 2010; Hong Kong,	147 811	45–84	51	Patients with T2DM, without CVD	SD	HR from multivariable Cox proportional hazard regressions	Cardiovascular disease (CVD) and mortality risk
Kim et al., 2021 [40]	Retrospective cohort study; –; South Korea	434	58 ± 10	54	Patients with T2DM	CV	HR form Cox proportional hazards regression analysis	Diabetic retinopathy
Lee S. et al., 2021 [41]	Single-centre, retrospective observational cohort study; Jan 2009–May 2019; Hong Kong	3424	60 ± 20	50	Patients with diabetes mellitus who were prescribed insulin at outpatient clinics	SD; CV	Odds ratio (OR) and hazard ratio (HR) for logistic and Cox regression	All-cause mortality, multiple complications including cardiovascular mortality, stroke and micro-vascular complications
Shen et al., 2021 [42]	Retrospective cohort study; Jan 213–April 2018; USA	29 260	66 ± 12	46	Patients with T2DM of the Louisiana experiment assessing diabetes outcomes (LEAD) cohort study; 59% were whites and 41% African Americans	SD; CV	Hazard ratio (HR) from Cox regression models; multivariate-adjusted HRs calculated for first, second, third and fourth quartiles of HbA1c SD/CV	Incident cardiovascular disease
Ceriello et al., 2022 [43]	Retrospective cohort study on Swedish national diabetes register (NDR); 3y exposure + 5y longitudinal phase; Sweden	101 533	64 (52–72)	56	People with T2DM with at least five HbA1c measurements, in the NDR between January 1st, 2000, and September 25th, 2019	SD	Hazard ratio (HR) from multivariate Cox regression analyses, according to quartiles	Non-fatal myocardial infarction, non-fatal stroke, all-cause mortality, multiple complications
Wu et al., 2022 [44]	Prospective cohort study from diabetes shared care program; 2004–2015; Taiwan	1 869	63 ± 13	50	Patients with T2DM	SD	HR from Cox proportional hazard, 3 models	Diabetic nephropathy, retinopathy, all-cause mortality and CVD mortality
Yan et al., 2022 [45]	Clinic-based retrospective longitudinal study; Jul 1999–Oct 2019; Japan	699	56 + 10	68	Consecutive T2DM patients attending outpatient clinic	CV	HR from Cox proportional hazards models adjusted for cofounders, 3 models	Diabetic nephropathy

**Fig. 2:**
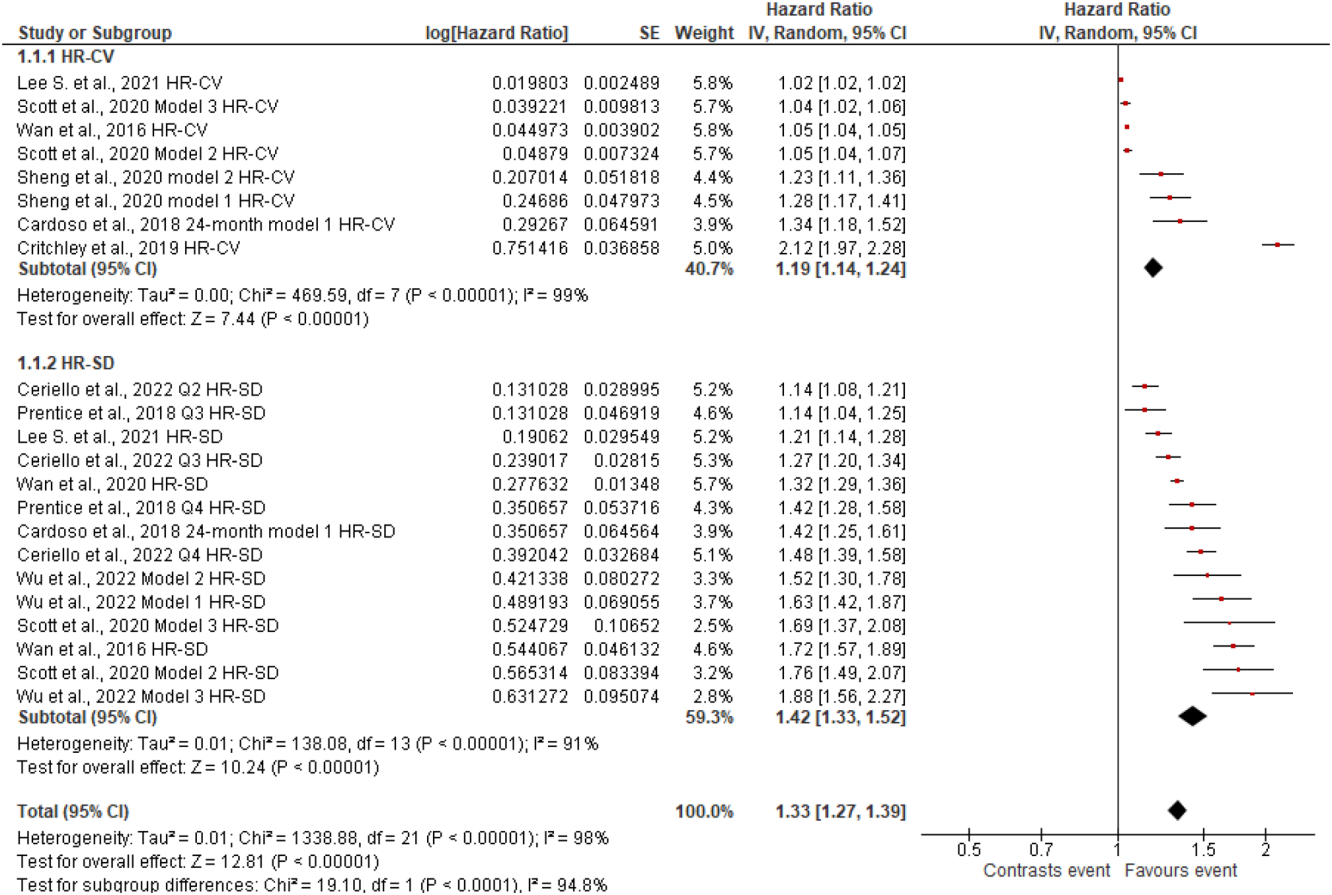
Forest plot of data regarding all-cause mortality, random-effect model. Hazard ratios (HR) for HbA1c-CV and HbA1c-SD, according to published available reports for T2DM. [*Note*: Here and in the following figures, each included study is represented by a point estimate of intervention effect, completed with a horizontal line extending either side (indicating the 95% confidence interval, 95%CI); the summary result is represented as a diamond at the bottom of each subgroup and as a final overall estimate.]

**Fig. 3:**
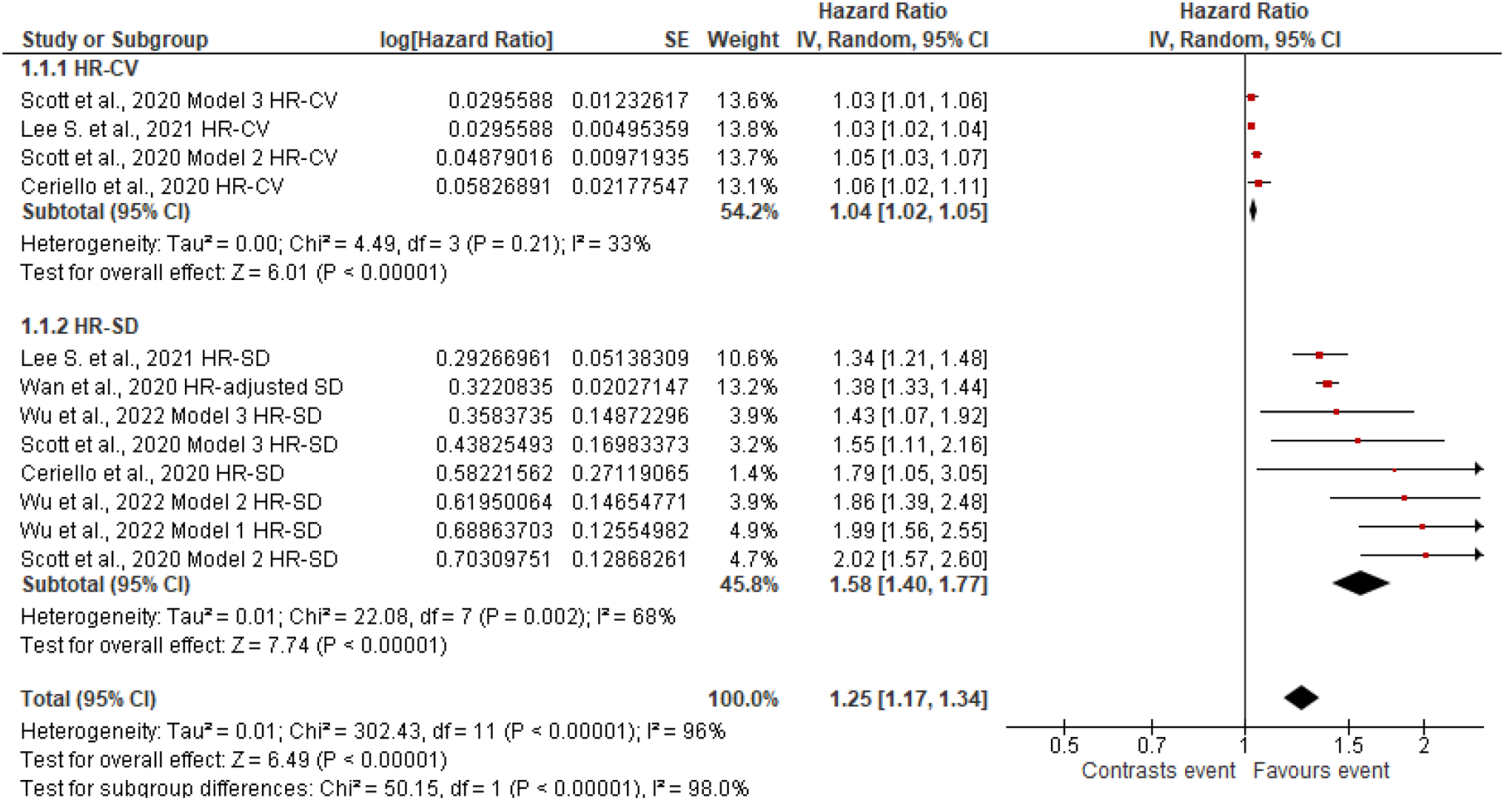
Forest plot of data regarding cardiovascular mortality, random-effect model. Hazard ratios (HR) for HbA1c-CV and HbA1c-SD, according to published available reports for T2DM

**Fig. 4:**
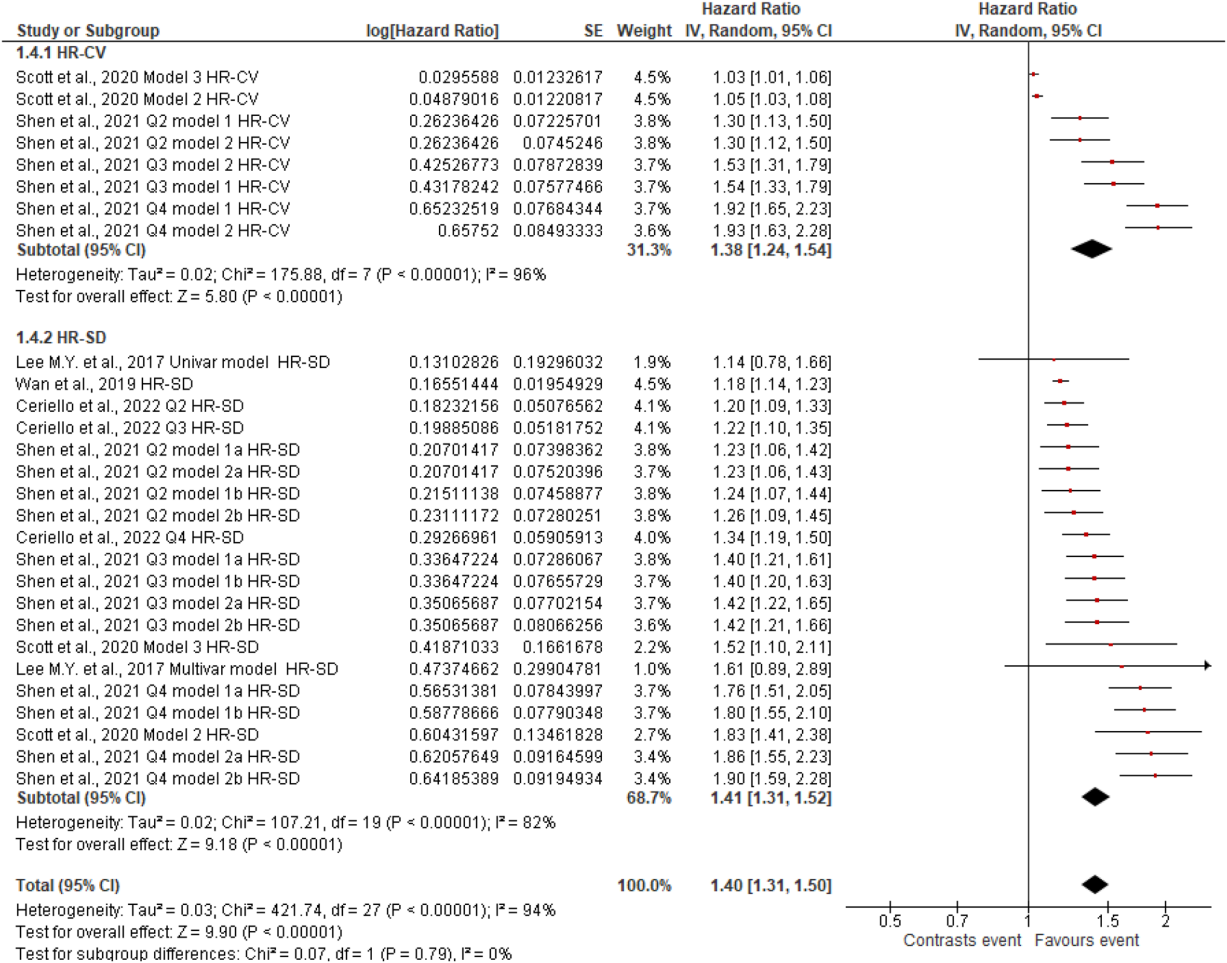
Forest plot of data regarding stroke, random-effect model. Hazard ratios (HR) for HbA1c-CV and HbA1c-SD, according to published available reports for T2DM

**Fig. 5:**
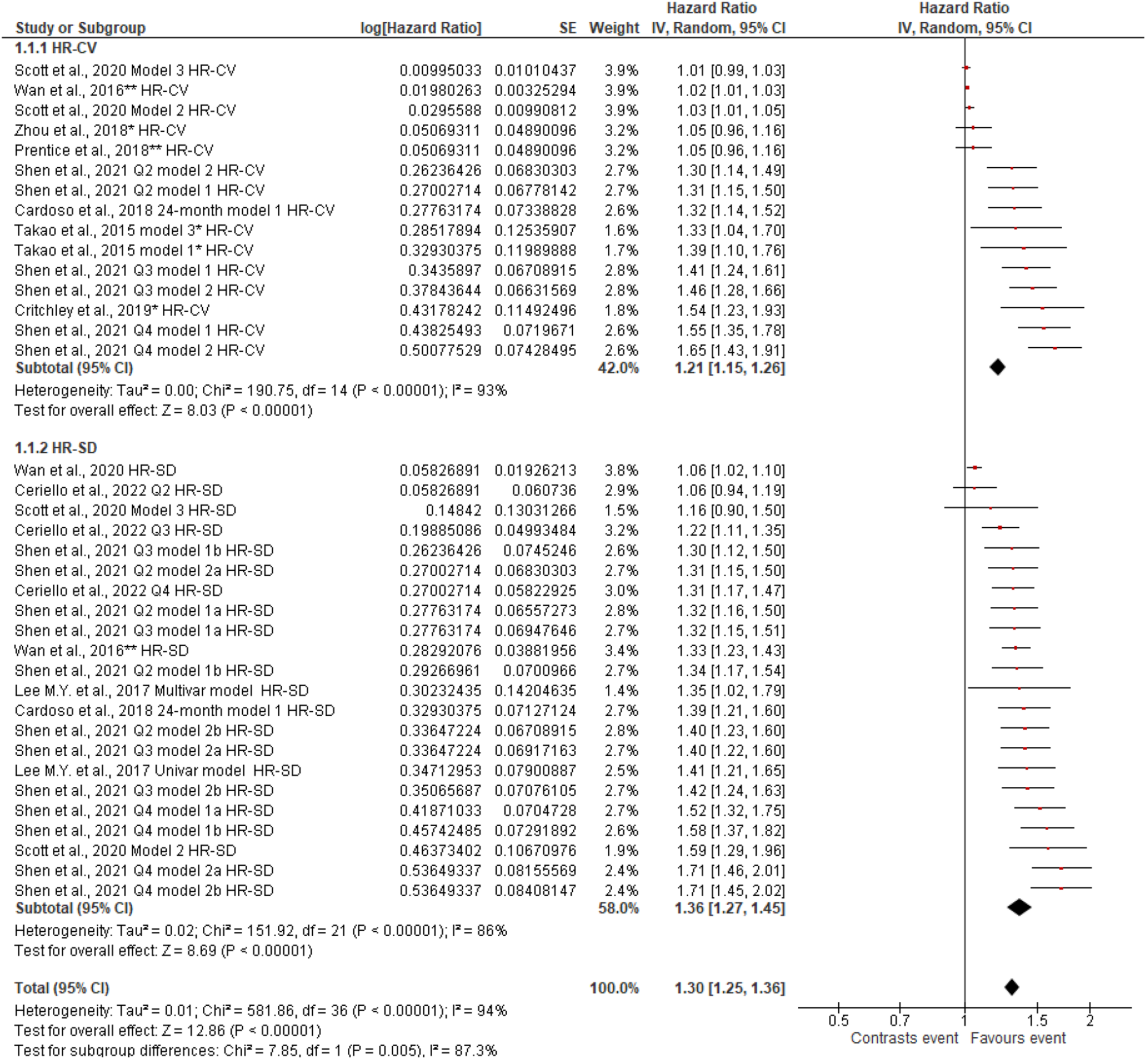
Forest plot of data regarding transient ischaemic attack (TIA), coronary heart disease (CHD), myocardial infarction (MI); random-effect model. Hazard ratios (HR) for HbA1c-CV and HbA1c-SD, according to published available reports for T2DM. *including stroke and death; **including stroke

**Fig. 6:**
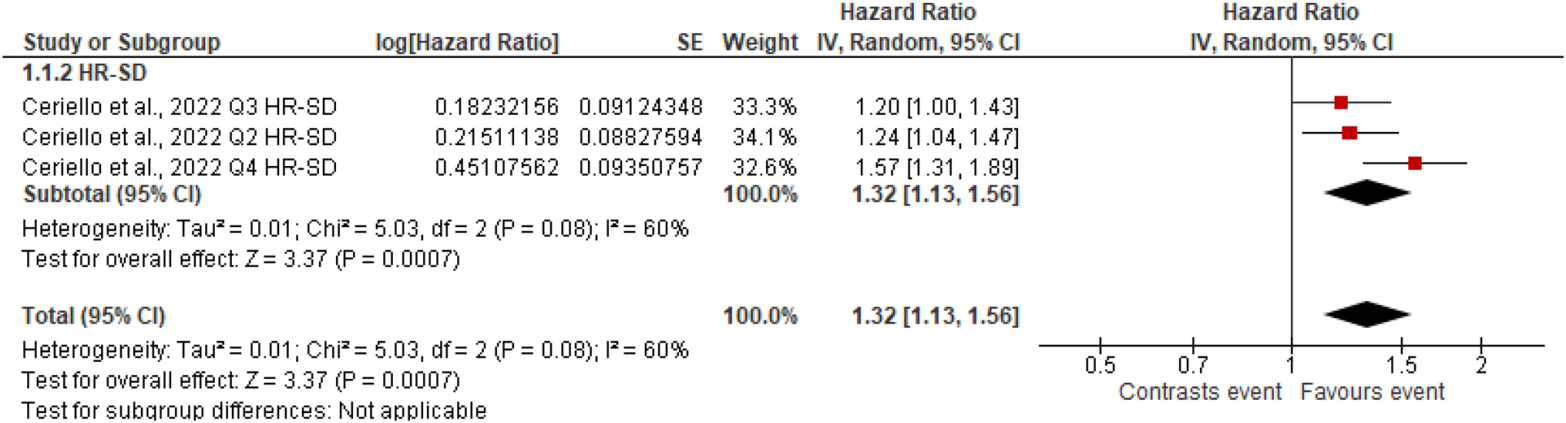
Forest plot of data regarding peripheral arterial disease, random effect model. Hazard ratios (HR) for HbA1c-SD, according to published available reports for T2DM

**Fig. 7:**
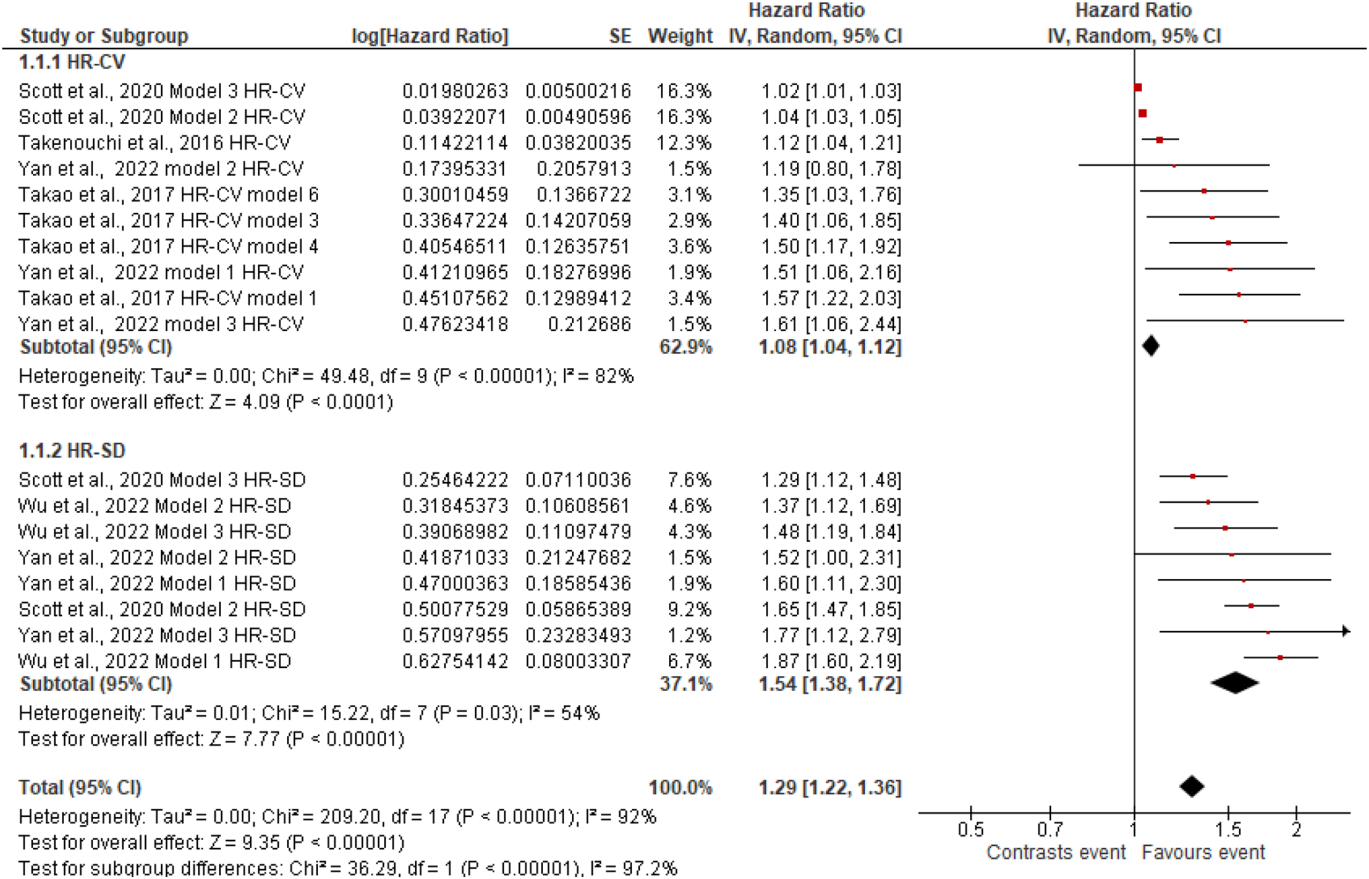
Forest plot of data regarding nephropathy, random-effect model. Hazard ratios (HR) for HbA1c-CV and HbA1c-SD, according to published available reports for T2DM

**Fig. 8:**
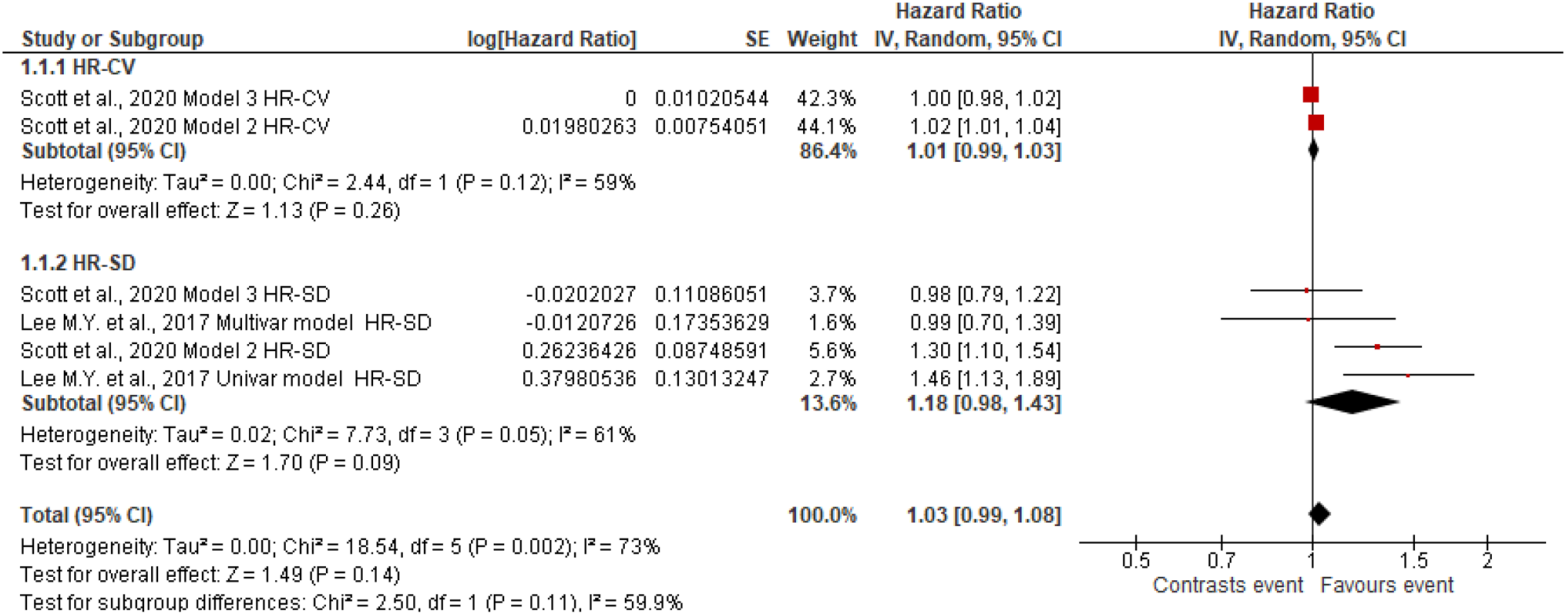
Forest plot of data regarding neuropathy, random-effect model. Hazard ratios (HR) for HbA1c-CV and HbA1c-SD, according to published available reports for T2DM

**Fig. 9:**
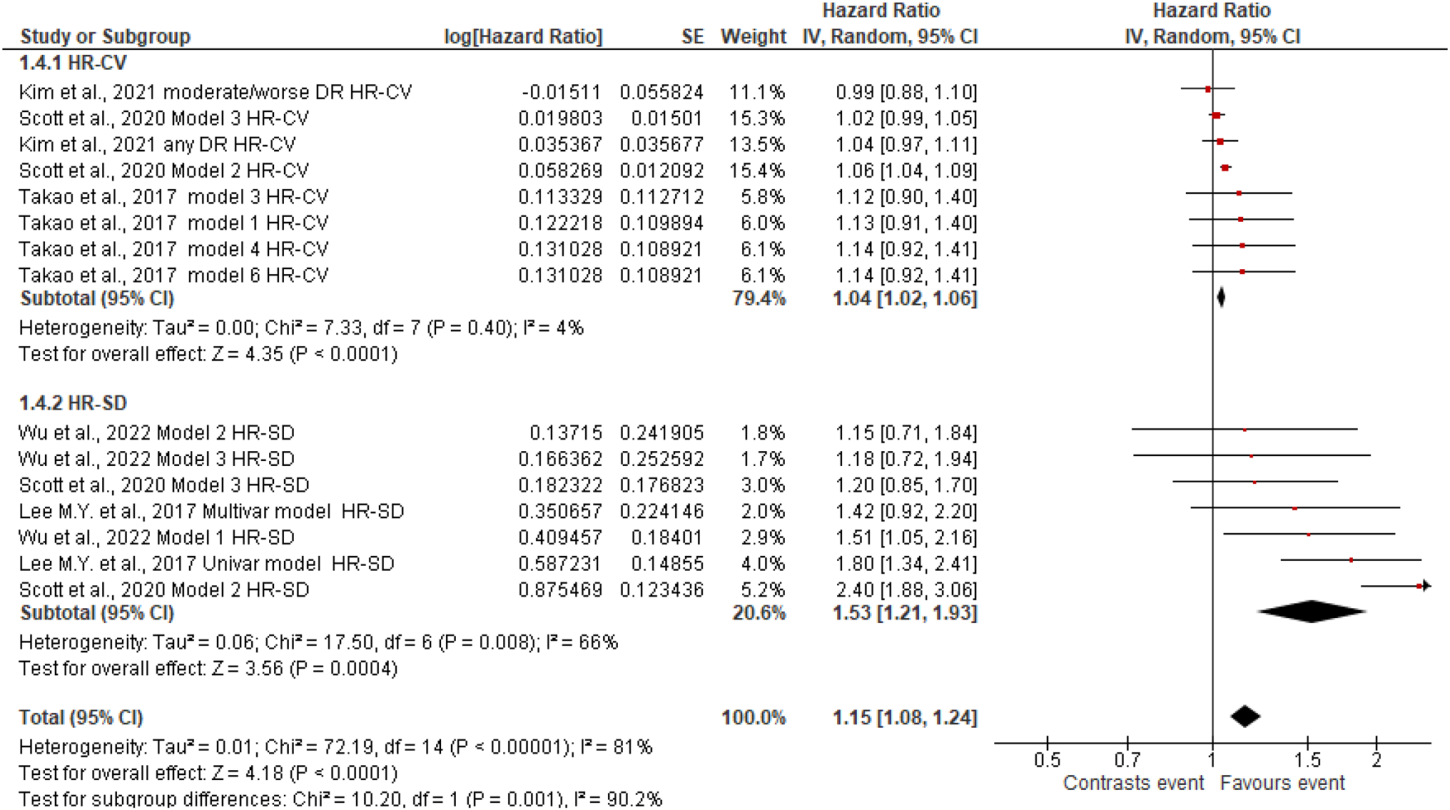
Forest plot of data regarding retinopathy, random-effect model. Hazard ratios (HR) for HbA1c-CV and HbA1c-SD, according to published available reports for T2DM

Figures 2, 3, 4, 5, 6, 7, 8 and 9 show the results of the meta-analysis as forest plots for the association between HbA1c variability and risk (evaluated as HR) of the various considered outcomes in people affected by T2DM. Overall, the analysis of the risk indicated that an association is appreciable between the HbA1c variability, expressed either as CV or as SD, and the various outcomes. The association appeared as statistically significant for all the considered complications (Figs. 2, 3, 4, 5, 6, 7, 9), except for neuropathy (Fig. 8). Averaged HR for all-cause mortality was 1.33 (95%CI 1.27–1.39, *p* < 0.0001), with the contributing effect of both HbA1c-CV and HbA1c-SD (Fig. 2), also confirmed for the data regarding cardiovascular mortality with a total HR of 1.25 (95%CI 1.17–1.34, *p* < 0.0001) (Fig. 3). Macro-vascular complications were all significantly associated with HbA1c variability parameter, either expressed as CV or SD, with an HR of 1.40 (95%CI 1.31–1.50, *p* < 0.0001) for stroke (Fig. 4), HR of 1.30 (95%CI 1.25–1.36, *p* < 0.0001) for transient ischaemic attack/coronary heart disease/myocardial infarction (Fig. 5), and 1.32 (95%CI 1.13–1.56, *p* = 0.0007) for peripheral arterial disease (Fig. 6). Considering micro-vascular complications, HR was 1.29 (95%CI 1.22–1.36, *p* < 0.0001) for nephropathy (Fig. 7), HR 1.03 (95%CI 0.99–1.08, *p* = 0.14) for neuropathy (Fig. 8) and HR 1.15 (95%CI 1.08–1.24, *p* < 0.0001) for retinopathy (Fig. 9). Since data coming from the studies show considerably different estimates of risk, the heterogeneity measure, provided in particular by the *I*^*2*^ statistics (see forest plot details in Figs. 2, 3, 4, 5, 6, 7, 8 and 9), indicates an appreciable and significant variability among studies, with ranges also greater than 50%.

**Fig. 10:**
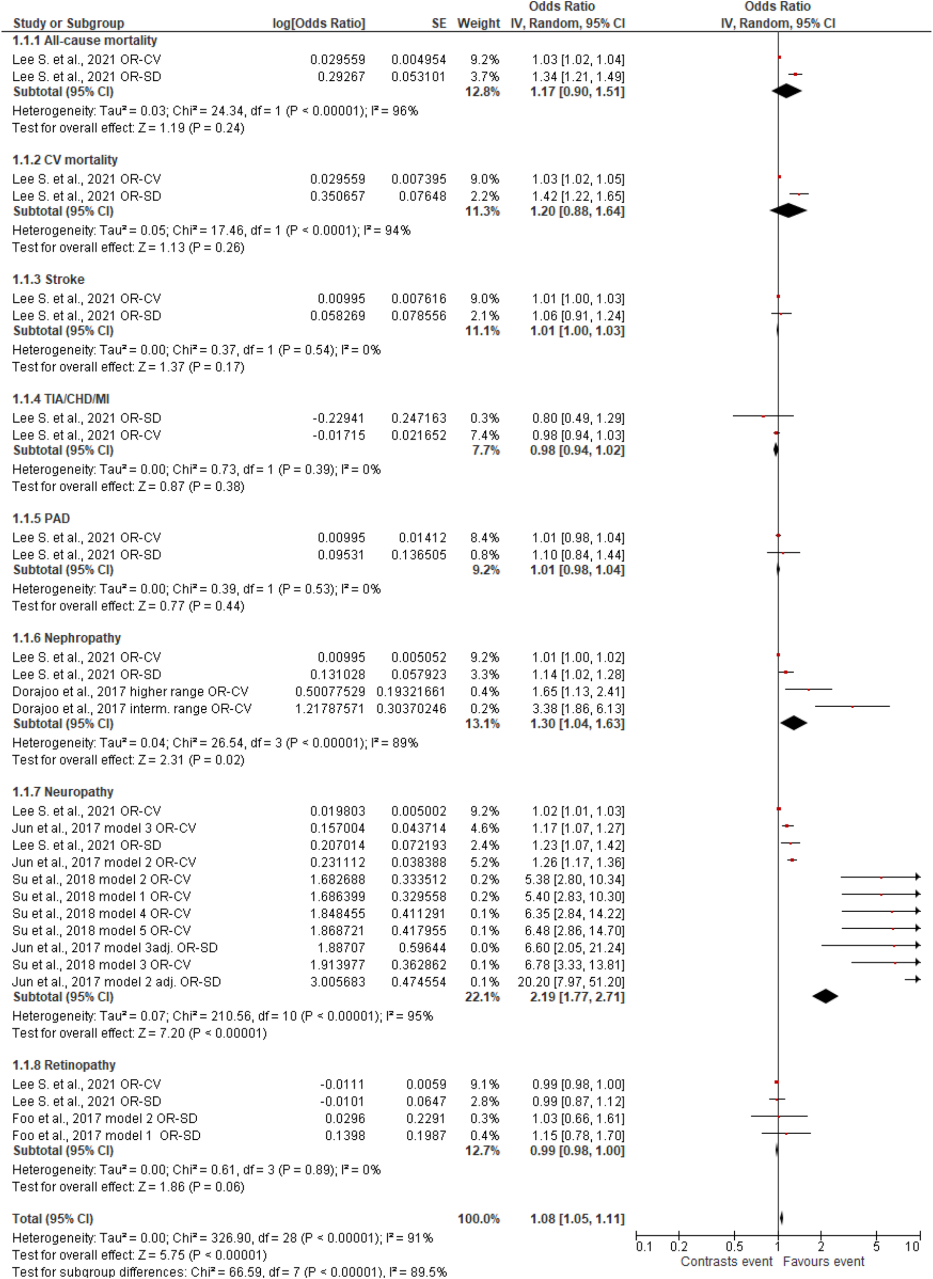
Forest plot of data regarding all risk factors, random-effect model. Odds Ratios (OR) for HbA1c-CV and HbA1c-SD, according to published available reports for T2DM. TIA/CHD/MI: transient ischaemic attack, coronary heart disease, myocardial infarction. PAD: peripheral arterial disease

Considering the OR as measure of association between HbA1c variability (expressed either as CV or SD) and risk for each of the outcomes (Fig. 10), most of these presented a non-significant association (for all-cause mortality, cardiovascular mortality, stroke, TIA/CHD/MI, PAD, retinopathy), while a significant association was found only for two micro-complications (namely nephropathy and neuropathy). However, the results regarding OR for these two micro-complications, in particular for data referred to neuropathy, are characterized by a high level of heterogeneity (Tau^2^ test and *I*^*2*^ statistics), which may affect each relative overall effect.

## Discussion

In this meta-analysis, we assessed the association and the potential clinical utility of HbA1c variability in T2DM, focusing on studies published in the last years, from 2015 until present day. The relevance of the issue consists of the fact that HbA1c variability could be a potentially modifiable risk factor and therefore could provide additional information for an optimized management of diabetes mellitus. The present analysis suggests that HbA1c variability (assessed as SD or CV) is significantly associated with the risk (as HR) of macro-vascular complications in T2DM, therefore conditioning also all-cause mortality and specifically cardiovascular mortality. On the other side, HbA1c variability may predict a significant risk of micro-vascular complications only for nephropathy and retinopathy, while the neuropathy risk appears not relevant.

These results confirm that individualizing care on the basis of change in glycaemic variability can be an important aspect of precision medicine in diabetes managing [46], although such an objective might take a long time to be achieved. A critical point is also the choice of the optimal predictor parameter among those proposed for the control of diabetes. Glycaemic variability is usually defined by the measurement of fluctuations of glucose or other related parameters of glucose homeostasis over a given interval of time. It can be measured over the short-term glycaemic variability with continuous glucose monitoring (CGM) of interstitial fluid glucose levels both within-day and between-day, and long-term glycaemic variability with the oscillation of fasting plasma glucose (FPG) or HbA1c levels over a longer period, usually months or years [38]. Published results suggested a role of CGM as risk factor for retinopathy, regardless of HbA1c [47]. Most previous studies of subjects with T2DM considered FPG variability as an indicator of glycaemic variability; however, FPG has a limitation in that it does not reflect postprandial glucose levels, which is considered important in terms of diabetes control. HbA1c can be better indicator in that it reflects both FPG and postprandial glucose levels [48]. In this review, we choose HbA1c variability (intended as SD and/or CV) as a parameter of long-term glycaemic variability. Despite the emerging evidence for the predictive value of HbA1c variability, its clinical application remains limited by the absence of a standardized quantification method [41].

The burden of HbA1c variability found by the present meta-analysis in patients affected by T2DM regarding vascular complications appears to have approximately the same weight as the main detected risk factors, such as age, male sex and history of hypoglycaemia [49], suggesting that HbA1c variability can have a greater impact on the development of complications than the HbA1c level per se. From this observation, it follows that in order to prevent diabetes-related complications it is advisable to consider the variability of HbA1c among the reference parameters for patient follow-up and to use hypoglycaemic therapies that can guarantee the greatest possible stability of this parameter over time. The new drugs seem to work in this direction; a recently concluded trial demonstrated that the sodium-glucose co-transporter 2 (SGLT2) inhibitor empagliflozin is associated with a reduced HbA1c variability [37], although the observed reduction in cardiovascular death following drug treatment did not appear to be mediated by reductions in HbA1c variability. However, in real-world setting, HbA1c variability in patients receiving a stable drug therapy, such as sulphonylureas, often appears large and not adequate to indicate the effectiveness of the treatment [50, 51], therefore limiting any predictive role of this glycaemic parameter on complication development.

As regard neuropathy, the HbA1c variability, assessed in the present analysis according to data on HR (Fig. 8), appeared to have a lower weight as risk predictor of complications in T2DM, not reaching a significant threshold. A published meta-analysis on potential risk factor for diabetic peripheral neuropathy had identified several risk factors associated, and among these is HbA1c [52], but also duration of diabetes, age and diabetic retinopathy. Other possible risk factors for neuropathy previously investigated are smoking, body mass index, serum lipid profile, but all of these were not found as significant predictors by Liu et al. [52], suggesting a still incomplete knowledge of the pathogenesis of this micro-vascular complication. On the other hand, it could be hypothesized that short-term glycaemic variability could play a greater role than a long-term variability in affecting axonal degeneration, contributing to the development of diabetic peripheral neuropathy [53]. Moreover, most published data considering diabetic peripheral neuropathy consist of cross-sectional studies with limited sample size [52], therefore affecting a low statistical power. In the present analysis, the association of HbA1c variability with other diabetic micro-complications, namely nephropathy and retinopathy, reached the significant threshold; this fact probably is linked also to the fact that the available data in the literature are more abundant for these two complications compared to neuropathy, and mostly in favour of the risk of vascular damage, therefore conditioning a significant overall effect. It must be considered, as indicated in the Methods section, that when an article presented more estimates of the risks linked to HbA1c variability, based on different mathematical models, all the available proposed data were considered for the meta-analysis, in order to have a wider view on the associations with risks of complications. This fact may have induced an appreciable effect on the statistical significance of the overall effect. Furthermore, it should be noticed the occurrence of an overall stronger HR for HbA1c variability linked to macro-vascular complications, compared to micro-vascular complications; probably, this finding could be related to the role of impaired glucose homeostasis on lipid abnormalities. In fact, it is known that people affected by T2DM and characterized by insulin resistance, often present atherogenic dyslipidaemia, which is a key causal factor linked to the development of atherosclerosis [54]. It is possible to suggest that further studies should evaluate the role of other factors, such as AGEs, ROS and in general oxidative stress, as possible determinants involved in the diabetic peripheral neuropathy as well as in the other micro-vascular complications of T2DM, potentially with different role on pathogenicity to the target tissue.

Still regarding neuropathy, the analysis of data set providing estimates of HbA1c variability in terms of OR (Fig. 10) permitted to observe a significant overall effect. This fact could be related to the presence of quite large estimates of OR obtained through various risk models from two studies [12, 31]; however, the authors noticed that a selection bias might have occurred, linked to the retrospective design and the origin of patients from a tertiary hospital [31], or the lack of any evaluation of the possible role of indices of oxidative stress, inflammation or endothelial dysfunction [12].

The results of the meta-analysis considering the predictive role of HbA1c on various vascular complications (Figs. 2, 3, 4, 5, 6, 7, 8, 9 and 10) show also that the risk calculated for CV and SD has a different power, being values for CV lower than those for SD. This difference depends on the mean HbA1c values and suggests that in people with poorly controlled diabetes with high mean HbA1c the CV will be low, whereas in well controlled patients with the same variability (expressed as calculated SD) the CV will be higher. It may arise the question if there could be a different effect of the similar variability calculated by SD in patients with well or poorly controlled diabetes on development of complications and whether the predictive value of high HbA1c variability is different in patients with differently controlled diabetes. The answer to this question is not univocal, since the published studies differ in their choice of reference point and thereby in the interpretation of the measure; so, there is a clear need to define a unique parameter for the variability of HbA1c, which has statistical value and is easily applicable in clinical practice. The difficulty in the definition and interpretation of HbA1c variability has been already noted by several authors [55, 56], and it is unclear which index of visit-to-visit variability of HbA1c is most useful for predicting the risks taken into account, or there may be other useful indices. In order to clarify the causal relationship between visit-to-visit variability of HbA1c and the risks, comparative studies using various indices should be performed.

The present meta-analysis revealed different performance in defining the risk associated with HbA1c considering either HR or OR. It should be noted that OR indicates the presence of an association between an intervention and related risk, summarizing an overall study, but tends to exaggerate risk; moreover, the OR is a static measure and does not consider rates. Conversely, HR considers rates and indicates how the intervention modifies the rate of experiencing the considered event. HR, generally obtained with the standard Cox regression method, permits to investigate the effect of one or more variables (covariates) on the “time-to-first-event” [57]. Therefore, HR gives information on a phenomenon over time [58] and can be considered the representation of instantaneous risk. Moreover, OR tends to overestimate the risk, if compared to HR [59]. This fact has been encountered in the present meta-analysis, such as for the above-mentioned neuropathy event. Curiously, considering the evaluated complications of which available data regarding OR linked to HbA1c variability have been found in the literature and therefore here considered in the meta-analysis, most of the associations with mortality or macro-vascular complications did not result as significant (Fig. 10). Possible influence of OR as a static measure might have contributed to this result, as well as the very limited number of available published studies.

Regarding the heterogeneity issue for HR data, the considered outcomes present an appreciable variability among studies, since the heterogeneity measure, in particular as *I*^*2*^ statistics, very often presents a significant value, with ranges also greater than 50%, suggesting substantial/considerable heterogeneity [23]. Since the random-effect method of analysis was used, the results pertain to the mean effects across studies, indicating a considerable discrepancy among published studies. However, inspection of the data distribution in the forest plot suggests that the majority of studies, although characterized by an overall sustained heterogeneity, are mostly located beyond the line of null effect, suggesting an overall increase of the risk of the outcome. As already observed above, it is to be noted that the meta-analysis here conducted considered also different estimates (models) provided by the same study, therefore possibly contributing to enhance the data dispersion.

Concerning OR, the results for two micro-complications (nephropathy and neuropathy) are characterized by a relevant heterogeneity, as suggested by Tau^2^ and *I*^*2*^ parameters, and this fact may influence the overall effect. Moreover, data for neuropathy are influenced greatly by the presence of several values coming from a single study [12] obtained with different models for risk estimation on the same data of origin, suggesting a possible major influence of the calculation algorithms.

## Conclusion

Our meta-analysis on HbA1c variability performed on the most recent published data since 2015 extends the view of previously published study by Gorst et al. [15] and confirms the positive association between HbA1c variability and macro-/micro-vascular complications, as well as mortality events, in T2DM, suggesting that this long-term glycaemic parameter merits further attention as a predictive, independent risk factor for T2DM population.

## Supplementary Information

Below is the link to the electronic supplementary material.

Supplementary file1 (DOCX 19 KB)
