# Supplementary material for: Long-term HbA1c variability and macro-/micro-vascular complications in type 2 diabetes mellitus: a meta-analysis update
Source: Acta Diabetol. 2023 Jan 30;60(6):721–38. doi: 10.1007/s00592-023-02037-8 (PMC10148792; doi:10.1007/s00592-023-02037-8)
Supplement: Supplementary file 1 — Supplementary file1 (DOCX 19 KB) [file 592_2023_2037_MOESM1_ESM.docx]

Sartore et al., **Long-term HbA1c variability and macro-/micro-vascular complications in type 2 diabetes mellitus: a meta-analysis update**

**Supplementary Information**

**Table S1.** Studies not included in the analysis (see diagram of figure 1). Papers are presented in chronological order of publication.

| **Study** | **Type of study; time interval considered; country** | **Sample size (n)** | **Age of patients (mean ± SD, or median & range)** | **Gender (Male %)** | **Inclusion criteria** | **Measure of HbA1c variability** | **Methodological notes** | **Evaluated complications** |
| --- | --- | --- | --- | --- | --- | --- | --- | --- |
| Bonke et al., 2016 [1] | Retrospective study on routine data collected to monitor the Bavarian Disease Management Program (DMP); Oct 2003 - Dec 2013; Germany | 13 777 | 67±11 | 55 | Patients with T2DM | mean of the absolute HbA1c differences | Cox regression models | Non-fatal CVD event |
| Laiteerapong et al., 2017 [2] | Longitudinal follow-up from Kaiser Permanente Northern California Diabetes Registry; 1997-2001; USA | 28 016 | 48-57 | 56 | Patients with T2DM | 10-year HbA1c trajectory | HR from Cox proportional hazard models | Microvascular and macrovascular events and mortality. |
| Low et al., 2017 [3] | Retrospective study; 2002-2014; Singapore | 1628 | --- | 58 | Patients with T2DM attending diabetes centre | CV | OR from multivariable logistic regression, different models for quartiles and medianes | Diabetic nephropathy, eGFR decline |
| Lee M.Y. et al., 2018 [4] | Prospective cohort study; 2007-2015; Taiwan | 388 | 66±11 | 60 | Consecutively recruited patients with T2DM and evidence of kidney damage, from a regional hospital | SD | HR from Cox proportional hazard model | Diabetic nephropathy, progression to dialysis |
| Matsutani et al., 2018 [5] | Retrospective study; 2 y; Japan | 57 | 67±8 | 68 | Patients with T2DM who underwent  CGM and BRS evaluations | SD; CV | --- | Baroreflex sensitivity (BRS) |
| Echouffo-Tcheugui et al., 2019 [6] | Prospective cohort analysis on the multicenter Antihypertensive and Lipid-Lowering Treatment to Prevent Heart Attack Trial (ALLHAT); 1994-2006; USA, Canada, Puerto Rico | 4 982 | 65 (60-70) | 57 | Patients from ALLHAT multicenter trial of hypertension therapy, including individuals with or without diabetes | (Glycaemia CV) | HR from multivariable Cox proportional hazards regression models | Incident CVD (coronary heart disease [CHD], stroke, and heart failure [HF]) and all-cause mortality |
| Lai et al., 2019 [7] | Perspective cohort study; 67 months mean follow up; Taiwan | 223 | 63 | 65 | T2DM patients | SD; CV | Composite scores for severity of disease | Diabetic nephropathy |
| Lee C.L. et al., 2020 [8] | Prospective cohort study; 2006-2011; Taiwan | 1 383 | 66±13 | 52 | Patients with T2DM recruited from outpatient department at a medical center | CV | Multivariable adjusted annual decline of eGFR | Diabetic nephropathy |
| Li Sh. et al., 2020 [9] | Retrospective cohort study on patients from Tayside and Fife in the Scottish Care Information–Diabetes Collaboration (SCI-DC); -- ; Scotland, UK | 13 111–19 883 | 63±11 | 56 | Patients with newly diagnosed T2DM | HVS (HbA1c variability score) | Cox proportional hazards model to assess the association between HbA1c variability and each of the outcomes | All-cause mortality, cardiovascular events, and microvascular complications |
| Li Su.et al., 2020 [10] | Prospective, longitudinal cohort study; Jan 2013-Dec 2014; China | 466 | 59±12 | 48 | Patients with T2DM with normal cardiac structure and function | SD | no data on HR or OR | Left ventricular remodeling |
| Segar et al., 2020 [11] | Multicenter clinical study “Action to Control Cardiovascular Risk in Diabetes” (ACCORD) trial; 3 y; USA, Canada | 8576 | 63±6 | 62 | Patients with T2DM enrolled in the Action to Control Cardiovascular Risk in Diabetes (ACCORD) trial | SD; CV | Hazard ratio (HR) from Cox regression models | Heart failure (HF) |
| Mao et al., 2022 [12] | prospective Hong Kong Diabetes Register cohort; 1995-2019; Hong Kong | 15 286 | 61±11 | 52 | Patients with T1DM & T2DM | HVS | HR from multivariate Cox regression models adjusted for confounders | All-site cancer (primary outcome) and cause-specific death (secondary outcome). |

**References**

1. Bonke FC, Donnachie E, Schneider A, Mehring M (2016) Association of the average rate of change in HbA1c with severe adverse events: a longitudinal evaluation of audit data from the Bavarian Disease Management Program for patients with type 2 diabetes mellitus. Diabetologia 59(2):286-293. https://doi.org/10.1007/s00125-015-3797-z

2. Laiteerapong N, Karter AJ, Moffet HH, Cooper JM, Gibbons RD, Liu JY, Gao Y, Huang ES (2017) Ten-year hemoglobin A1c trajectories and outcomes in type 2 diabetes mellitus: The Diabetes & Aging Study. J Diabetes Complications 31(1):94-100. https://doi.org/10.1016/j.jdiacomp.2016.07.023

3. Low S, Lim SC, Yeoh LY, Liu YL, Liu JJ, Fun S, Su C, Zhang X, Subramaniam T, Sum CF (2017) Effect of long-term glycemic variability on estimated glomerular filtration rate decline among patients with type 2 diabetes mellitus: Insights from the Diabetic Nephropathy Cohort in Singapore. J Diabetes 9(10):908-919. https://doi.org/10.1111/1753-0407.12512

4. Lee MY, Huang JC, Chen SC, Chiou HC, Wu PY (2018) Association of HbA1C Variability and Renal Progression in Patients with Type 2 Diabetes with Chronic Kidney Disease Stages 3⁻4. Int J Mol Sci 19(12):4116. https://doi.org/10.3390/ijms19124116

5. Matsutani D, Sakamoto M, Minato S, Kayama Y, Takeda N, Horiuchi R, Utsunomiya K (2018) Visit-to-visit HbA1c variability is inversely related to baroreflex sensitivity independently of HbA1c value in type 2 diabetes. Cardiovasc Diabetol 17(1):100. doi: 10.1186/s12933-018-0743-7

6. Echouffo-Tcheugui JB, Zhao S, Brock G, Matsouaka RA, Kline D, Joseph JJ (2019) Visit-to-Visit Glycemic Variability and Risks of Cardiovascular Events and All-Cause Mortality: The ALLHAT Study. Diabetes Care 42(3):486-493. https://doi.org/10.2337/dc18-1430

7. Lai YR, Chiu WC, Huang CC, Tsai NW, Wang HC, Lin WC, Cheng BC, Su YJ, Su CM, Hsiao SY, Lu CH (2019) HbA1C Variability Is Strongly Associated with the Severity of Peripheral Neuropathy in Patients With Type 2 Diabetes. Front Neurosci 13:90. https://doi.org/10.3389/fnins.2019.00090

8. Lee CL, Chen CH, Wu MJ, Tsai SF (2020) The variability of glycated hemoglobin is associated with renal function decline in patients with type 2 diabetes. Ther Adv Chronic Dis 11:2040622319898370. https://doi.org/10.1177/2040622319898370

9. Li Sh, Nemeth I, Donnelly L, Hapca S, Zhou K, Pearson ER (2020) Visit-to-Visit HbA1c Variability Is Associated with Cardiovascular Disease and Microvascular Complications in Patients With Newly Diagnosed Type 2 Diabetes. Diabetes Care;43(2):426-432. https://doi.org/10.2337/dc19-0823

10. Li Su, Zheng Z, Tang X, Zhong J, Liu X, Zhao Y, Chen L, Zhu J, Liu J, Chen Y (2020) Impact of HbA1c variability on subclinical left ventricular remodeling and dysfunction in patients with type 2 diabetes mellitus. Clin Chim Acta 502:159-166. https://doi.org/10.1016/j.cca.2019.12.006

11. Segar MW, Patel KV, Vaduganathan M, Caughey MC, Butler J, Fonarow GC, Grodin JL, McGuire DK, Pandey A (2020) Association of Long-term Change and Variability in Glycemia with Risk of Incident Heart Failure Among Patients with Type 2 Diabetes: A Secondary Analysis of the ACCORD Trial. Diabetes Care 43(8):1920-1928. https://doi.org/10.2337/dc19-2541

12. Mao D, Lau ESH, Wu H, Yang A, Shi M, Fan B, Tam CHT, Chow E, Kong APS, Ma RCW, Luk A, Chan JCN (2022) Risk associations of long-term HbA1c variability and obesity on cancer events and cancer-specific death in 15,286 patients with diabetes - A prospective cohort study. Lancet Reg Health West Pac 18:100315. https://doi.org/10.1016/j.lanwpc.2021.100315
